# Maternal Early Pregnancy Serum Metabolomics Profile and Abnormal Vaginal Bleeding as Predictors of Placental Abruption: A Prospective Study

**DOI:** 10.1371/journal.pone.0156755

**Published:** 2016-06-14

**Authors:** Bizu Gelaye, Susan J. Sumner, Susan McRitchie, James E. Carlson, Cande V. Ananth, Daniel A. Enquobahrie, Chunfang Qiu, Tanya K. Sorensen, Michelle A. Williams

**Affiliations:** 1 Department of Epidemiology, Harvard *T*.*H*. *Chan* School of Public Health, Boston, MA, United States of America; 2 Discovery Sciences, RTI International, Research Triangle Park, NC, United States of America; 3 Department of Obstetrics and Gynecology, College of Physicians and Surgeons, Columbia University, New York, NY, United States of America; 4 Department of Epidemiology, *Joseph L*. *Mailman* School of Public Health, Columbia University, New York, NY, United States of America; 5 Department of Epidemiology, School of Public Health, University of Washington, Seattle, WA, United States of America; 6 Center for Perinatal Studies, Swedish Medical Center, Seattle, WA, United States of America; University of Pittsburgh, UNITED STATES

## Abstract

**Background & Objective:**

Placental abruption, an ischemic placental disorder, complicates about 1 in 100 pregnancies, and is an important cause of maternal and perinatal morbidity and mortality worldwide. Metabolomics holds promise for improving the phenotyping, prediction and understanding of pathophysiologic mechanisms of complex clinical disorders including abruption. We sought to evaluate maternal early pregnancy pre-diagnostic serum metabolic profiles and abnormal vaginal bleeding as predictors of abruption later in pregnancy.

**Methods:**

Maternal serum was collected in early pregnancy (mean 16 weeks, range 15 to 22 weeks) from 51 abruption cases and 51 controls. Quantitative targeted metabolic profiles of serum were acquired using electrospray ionization liquid chromatography-mass spectrometry (ESI-LC-MS/MS) and the Absolute IDQ® p180 kit. Maternal sociodemographic characteristics and reproductive history were abstracted from medical records. Stepwise logistic regression models were developed to evaluate the extent to which metabolites aid in the prediction of abruption. We evaluated the predictive performance of the set of selected metabolites using a receiver operating characteristics (ROC) curve analysis and area under the curve (AUC).

**Results:**

Early pregnancy vaginal bleeding, dodecanoylcarnitine/dodecenoylcarnitine (C12 / C12:1), and phosphatidylcholine acyl-alkyl C 38:1 (PC ae C38:1) strongly predict abruption risk. The AUC for these metabolites alone was 0.68, for early pregnancy vaginal bleeding alone was 0.65, and combined the AUC improved to 0.75 with the addition of quantitative metabolite data (P = 0.003).

**Conclusion:**

Metabolomic profiles of early pregnancy maternal serum samples in addition to the clinical symptom, vaginal bleeding, may serve as important markers for the prediction of abruption. Larger studies are necessary to corroborate and validate these findings in other cohorts.

## Introduction

Placental abruption, the premature separation of the placenta from the uterus before delivery of the fetus, is a life threatening obstetrical event that complicates approximately 1% of pregnancies [1]. Abruption occurs more frequently among women with multifetal gestation, coagulopathies, acquired and heritable forms of thrombophilia, uterine anomalies, abdominal trauma, hypertension, premature rupture of membranes, maternal-fetal hemorrhage, evidence of mitochondrial dysfunction, oxidative stress, and intrauterine infections [2–8]. Other risk factors for abruption include advanced maternal age, grand-multiparity, maternal cigarette smoking and iron deficiency anemia during pregnancy [4–6,9]. Pathophysiologic mechanisms involved in abruption and related perinatal disorders (e.g., preterm delivery, preeclampsia, and intrauterine growth restriction) include uteroplacental ischemia, under perfusion, chronic hypoxia, and infarctions.

Investigators have explored a number of biomarkers related to the pathogenesis of abruption. These include maternal first or second trimester serum concentrations of alpha-fetoprotein, human chorionic gonadotropin, pregnancy associated plasma protein-A, CA 125, placental growth factor, soluble fms-like tyrosine kinase 1, endoglin and homocysteine [3,10–16]. Despite considerable effort, however, the etiology of abruption remains elusive. Furthermore, clinicians have little information to help support care decisions, particularly when patients present with signs and symptoms (e.g., vaginal bleeding in early pregnancy) of this potentially devastating complication.

Metabolomics, the systematic study of metabolites in tissues and biofluids, has emerged as a promising research tool to aid in building clinically relevant risk prediction models for disease classification and progression. In perinatal medicine, investigators have reported findings illustrating the promise that metabolomics holds in establishing detailed phenotypes of complex perinatal outcomes such as preterm delivery, fetal growth restriction preeclampsia, and gestational diabetes mellitus [17–20]. To our knowledge, no one has evaluated the extent to which metabolites measured in maternal pre-diagnostic (early pregnancy) biofluids differentiate women destined to develop abruption compared with those spared the disorder. To fill this important gap in the literature, we designed a case-control study, nested within a prospective cohort of pregnant women who provided serum samples in early pregnancy.

Specifically, we sought to establish the metabolic profiles of maternal sera from women with pregnancies complicated by abruption and a control group of women without abruption using targeted metabolomics methods. Our study has proposed biomarkers that, when validated in a larger cohort, may serve to identify women at risk for abruption, and may provide new clues to understand the pathogenesis of this devastating obstetrical complication.

## Material and Methods

### Study Cohort and Setting

A cohort of 4,009 pregnant women providing a second trimester serum sample to a central laboratory between 1994 and 1998, and who later delivered at Swedish Medical Center, WA constitute the base cohort population in which the present case-control study is nested. This study was part of the First and Second Trimester [9,21]. Each blood sample, collected at 15–22 weeks gestation (mean ± standard deviation (SD): 16 ± 1.6 weeks) was centrifuged, and serum was separated and stored at -20°C for 1 month. The serum was used to perform routine biochemical analyses as part of a second-trimester screening protocol for detecting pregnancies complicated by neural tube defects and Down’s syndrome [21]. Serum remaining after routine testing was transferred to the Center for Perinatal Studies at Swedish Medical Center and stored at -80°C. Aliquots were shipped to the National Institutes of Health funded Eastern Regional Comprehensive Metabolomics Resource Core at RTI International. All participants provided signed informed consent to use their medical records and blood samples left over after clinical tests were completed. This study was approved by the Institutional Review Board of Swedish Medical Center, WA.

### Placental abruption cases and controls

For this nested-case control study, we identified 51 women with clinically confirmed diagnosis of abruption for inclusion in the case group. For the purpose of the present study, we identified abruption cases through a careful review of medical records. We used medical records to identify patients with a clinical diagnosis of abruption and determine whether the diagnosis could be objectively supported by evidence of pathological characteristics, signs and symptoms of abruption. For the research diagnosis of abruption, using data abstracted from medical records, we required evidence of blood clot behind the placenta (specifically indicated in the delivery or pathology notes) accompanied by at least two of the following signs and symptoms: (i) vaginal bleeding in late pregnancy that was not associated with placenta previa or cervical lesions; (ii) uterine tenderness and/or abdominal pain; and (iii) fetal distress or death.

Women were eligible for inclusion in the study as controls if they had no clinical diagnosis of abruption in their medical records. We randomly selected 51 women who had no vaginal bleeding after 20 weeks gestation and who did not receive an abruption diagnosis. We abstracted data from medical records for all abruption cases and controls to ensure that antepartum and intrapartum medical histories are consistent with the presence (cases) or absence (controls) of abruption. All participants delivered singleton infants and none had an antepartum diagnosis of neural tube defect, Down’s syndrome or major congenital malformation.

Data on sociodemographic characteristics, reproductive and medical history, antenatal care and life-style characteristics were abstracted from medical records. Maternal abnormal early pregnancy vaginal bleeding (i.e., vaginal bleeding in the first or second trimester) was determined based on physician report indicated in maternal medical records (yes/no). Information about labor and delivery and infant outcomes was also collected from medical records. Confounding variables considered for adjustment included maternal age (<20, 20–34, ≥35 years), self-reported race/ethnicity (non-Hispanic White, African-American, Other), Medicaid payment status (yes/no), smoking during the index pregnancy (yes/no), chronic hypertension (yes/no), preeclampsia (yes/no), preterm delivery (<37 weeks), birthweight (<2500 g) (yes/no) and maternal pre-pregnancy body mass index (BMI).

### Metabolomics assays

Targeted metabolomics was conducted using electrospray ionization liquid chromatography–mass spectrometry (ESI-LC-MS/MS) and MS/MS and the Absolute*IDQ®* p180 kit (Biocrates Life Sciences AG, Innsbruck, Austria). This kit allowed for the simultaneous quantification of up to 188 metabolites, including free carnitine, 40 acylcarnitines (Cx:y), 21 amino acids (19 proteinogenic amino acids, citrulline and ornithine), 21 biogenic amines, hexose (sum of hexoses–about 90–95% glucose), 90 glycerophospholipids (14 lysophosphatidylcholines (lysoPC) and 76 phosphatidylcholines (PC diacyl (aa) and acyl-alkyl (ae)), and 15 sphingolipids (SMx:y). The assay details and the metabolite nomenclature for Absolute*IDQ®* p180 kit and have been previously described.[22] The stable labeled internal standard mixture and 10 μL of serum was applied directly to the sample well. Samples were derivatized and extracted in preparation for analysis. Mass spectrometric (MS) analyses were carried out on an API 4000 LC-MS/MS System (AB Sciex, Framingham, MA) equipped with an 1100 Series HPLC (Agilent Technologies, Palo Alto, CA) using an Agilent Eclipse XDB-C_18_ (3.5 μm) 3.0x100 mm column controlled by Analyst 1.6.2 software. A unique multiple reaction monitoring (MRM) pair, specific for each analyte, was used to measure the analytes and their stable labeled internal standard. The internal standard was used for absolute and/or relative quantification. All data were processed using a combination of Analyst 1.6.2 (AB Sciex LP, Ontario, Canada) and Met*IDQ* (Biocrates Life Sciences AG, Innsbruck, Austria) software.

Performance of quality control (QC) samples within an analytical run as well as pre- and post-analysis system suitability checks were assessed. QC measures include reviewing quality of each run, stability of internal standards signal, and drifts in retention time of standards. All laboratory analyses were completed without knowledge of participants’ case or control status. All raw and processed analytical data and associated de-identified metadata have been uploaded to the public accessible NIH Common Fund Metabolomics Data Repository (www.metabolomicsworkbench.org, Study ID#: ST000405).

### Statistical analysis

We used logistic regression modeling to evaluate the extent to which metabolites, both in isolation and combined with early pregnancy vaginal bleeding improves the prediction of abruption. Model selection was performed using stepwise logistic regression procedures (criteria: model entry P<0.1 and model removal P>0.2), continuous variables were centered, and the ratio of dodecanoylcarnitine/dodecenoylcarnitine (C12 / C12:1) was dichotomized at 1.2 (median value) and the final model was assessed for adequacy based on the Hosmer-Lemeshow goodness-of-fit test. Finally, we used the receiver operating characteristics (ROC) curve and the area under the curve (AUC) analysis to evaluate early pregnancy vaginal bleeding and metabolites as predictors of abruption. Statistical tests for metabolomics data were conducted using either a two-sided t-test with the Satterthwaite correction for unequal variances or the chi-square test. Statistical analyses for metabolomics data were conducted using SAS 9.4 (SAS Institute Inc., Cary, NC).

## Results

Maternal socio-demographic and clinical characteristics of abruption cases and controls are presented in **Table 1**. Cases and controls were similar as regards most maternal characteristics. Newborns delivered of women with abruption were more likely to be low birthweight and to be delivered preterm.

**Table 1:** Characteristics of Study Participants.

	Abruption cases	Controls	P-value***
	(N = 51)	(N = 51)	
	n (%)	n (%)	
Gestational Age at blood collection	16.2 ± 1.6*	16.1 ± 1.6	0.95
Maternal age (years)	30.9 ± 5.5*	31.0 ± 5.5	0.91
Maternal age (years)			
<20	2 (3.9)	1 (2.0)	0.63
20–34	33 (64.7)	37 (72.5)	
≥35	16 (31.4)	13 (25.5)	
Race			
Non-Hispanic White	31 (60.8)	41 (80.4)	0.12
African American	4 (7.8)	2 (3.9)	
Other	14 (27.5)	8 (15.7)	
Unknown	2 (3.9)	0 (0.0)	
Payment			
Insurance or private	38 (74.5)	36 (70.6)	0.79
Medicaid	9 (17.7)	12 (23.5)	
Unknown	4 (7.8)	3 (5.9)	
Nulliparous	23 (45.1)	22 (43.1)	0.99
Unmarried	11 (21.6)	9 (17.7)	0.62
Cigarette smoker during pregnancy	6 (11.8)	4 (7.8)	0.62
Alcohol consumed during pregnancy	7 (13.7)	11 (21.6)	0.44
Pre-pregnancy Body Mass Index (kg/m^2^)	24.0 ± 5.8*	24.0 ± 6.0	0.99
Vaginal bleeding in early pregnancy	18 (36.7)	5 (10.2)	0.02
Chronic hypertension	4 (7.0)	2 (3.9)	0.68
Preeclampsia	2 (100.0)	0(0.0)	—
Gestational age at delivery (weeks)	34.6 ± 5.1*	38.8 ± 2.0	<0.01
Preterm delivery (<37 weeks)	29 (56.9)	7 (13.7)	<0.01
Preterm PROM**	10 (19.6)	4 (7.8)	0.084
Male infant gender	31 (60.8)	26 (51.0)	0.32
Infant weight (grams)	2519 ± 1035*	3405 ± 552	<0.01
Low birth weight (<2500 g)	21 9 (41.2)	2 (3.9)	<0.01

*Mean ± SD (standard deviation)

** Premature rupture of membranes (PROM)

***P-value for Student’s *t* test or Chi-Square/Fisher’s Exact test)

**Fig 1:**
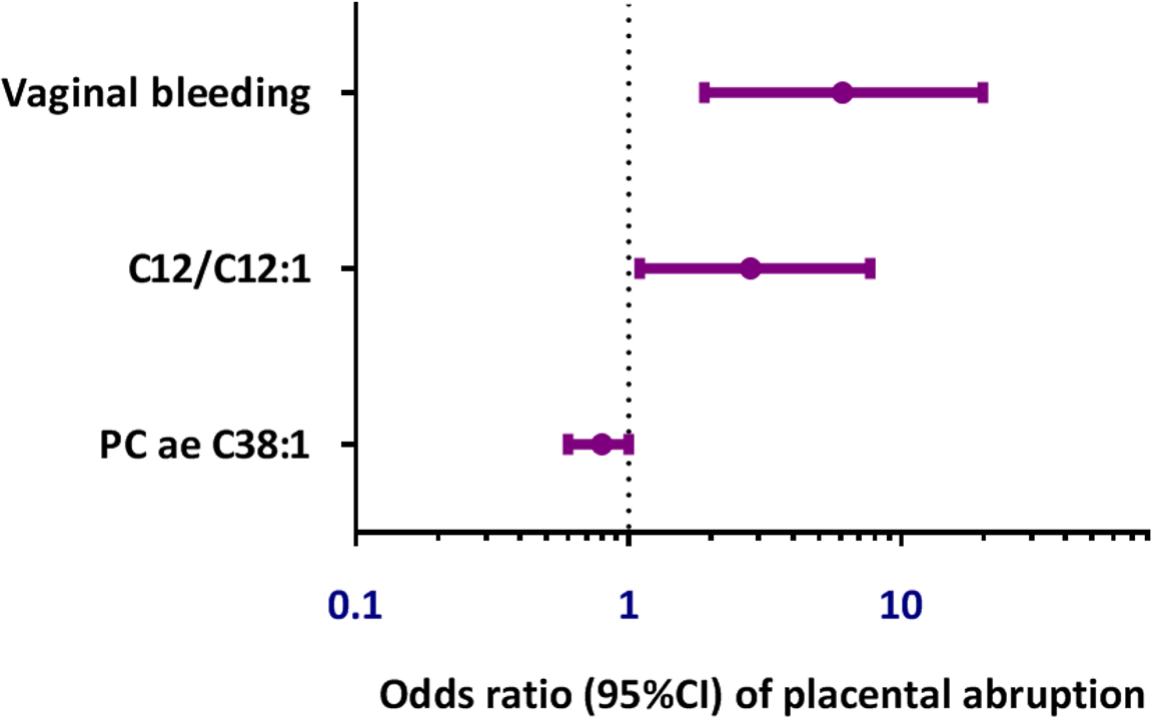
The odds ratio of placental abruption determined by a stepwise logistic regression model for metabolites associated with the occurrence of placental abruption and maternal history of abnormal early pregnancy vaginal bleeding.

Quantitative analyses of the 188 endogenous metabolites included in The Absolute*IDQ®* p180 kit (Biocrates Life Sciences AG, Innsbruck, Austria) indicated that 9 metabolites differentiated abruption cases as compared with controls. These metabolites included acylcarnitines, amino acids, amines, and glycerophopholipids (**Table 2**). Stepwise logistic regression modeling was used to identify metabolites associated with the occurrence of abruption. Candidate predictors were early pregnancy vaginal bleeding (yes/no) that was associated with abruption (P = 0.002), as well as compounds or custom ratios from the Biocrates analysis (P<0.05). Based on these model parameters, early pregnancy vaginal bleeding, dodecanoylcarnitine/dodecenoylcarnitine (C12 / C12:1), and phosphatidylcholine acyl-alkyl C 38:1 (PC ae C38:1) were selected, and the model fit the data with a Hosmer-Lemeshow goodness of fit of P = 0.17. The odds ratios determined by the stepwise logistic regression model are shown in **Fig 1**. The odds of abruption is increased with an increase in dodecanoylcarnitine/dodecenoylcarnitine (C12 / C12:1), and a decrease in phosphatidylcholine acyl-alkyl C 38:1 (PC ae C38:1).

**Table 2:** Metabolites that significantly differentiated placental abruption cases and controls via quantitative targeted analyses.

Metabolite		Limit of Detection	Abruption cases	Controls	Fold *	
Classification	Analyte	(LOD)	(N = 51)	(N = 51)	change	P-value**
			Mean ± SD	Mean ± SD		
Acylcarnitines	C16-OH	0.016	0.015 ± 0.0038	0.017 ± 0.0042	-1.12	0.021
Amino Acids	Arg^†^	1.64	197.63 ± 71.55	233.84 ± 90.99	-1.18	0.029
Biogenic Amines	Histamine	1.04	0.37 ± 0.01	0.37 ± 0.01	+1.01	0.034
Custom Ratios***	C12 / C12:1	C12 LOD: 0.09	1.16 ± 0.19	1.09 ± 0.19	+1.07	0.045
		C12:1 LOD: 0.066				
Glycerophospholipids	PC ae C38:1	0.033	5.71 ± 1.61	6.63 ± 2.29	-1.16	0.021
Glycerophospholipids	PC aa C40:1	0.472	0.56 ± 0.16	0.64 ± 0.22	-1.14	0.038
Glycerophospholipids	PC aa C36:0	0.273	1.91 ± 0.78	2.33 ± 1.18	-1.22	0.040
Glycerophospholipids	lysoPC a C18:1	0.183	15.54 ± 7.76	18.96 ± 8.76	-1.22	0.040
Glycerophospholipids	PC aa C38:0	0.047	2.03 ± 0.71	2.34 ± 0.84	-1.15	0.048
Custom Ratios***	High C12/C12:1	C12 LOD: 0.09	25 (49.02%)††	14 (27.45%)††	**—**	0.025
	Low C12/C12:1	C12:1 LOD: 0.066	26 (50.98%)††	37 (72.55%)††	**—**	

High C12/C12:1 = C12 / C12:1 > 1.2; Low C12/C12 = C12:1 ≤ 1.2

*Fold change (placental abruption case/control)

**p-values based on Student t tests with Satterthwaite approximation for continuous variables and chi-square test for categorical variables

***Ratio of dodecanoylcarnitine/dodecenoylcarnitine (C12 / C12:1)

^†^n = 50

^††^Cell count (%)

**Fig 2:**
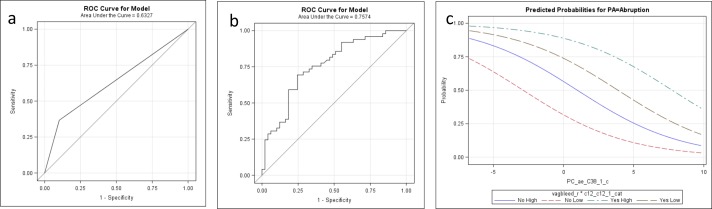
Receiver operating characteristic (ROC) curves for placental abruption prediction using (a) early pregnancy vaginal bleeding, (b) metabolites and early pregnancy vaginal bleeding, and (c) predicted probabilities for placental abruption.

We next assessed the extent to which maternal history of early pregnancy vaginal bleeding alone and combined with metabolomics data are predictive of later abruption. As shown in **Fig 2 and Table 3**, the AUC for early pregnancy vaginal bleeding alone was 0.63 (95% CI 0.55, 0.71). The AUC for the model with only the metabolite data was 0.68 (95% CI 0.58, 0.79) (not shown). Notably the AUC for bleeding combined with the quantitative metabolite biomarkers improved to 0.76 (95% CI 0.66, 0.85) (P for model differences = 0.003). The predicted probabilities for the abruption model including early pregnancy vaginal bleeding and metabolite data are shown in the right panel of **Fig 2**. Consistent with the **Fig 1**, the probability of abruption is increased with the presence of vaginal bleeding and an increase in dodecanoylcarnitine/dodecenoylcarnitine (C12 / C12:1), and a decrease in phosphatidylcholine acyl-alkyl C 38:1 (PC ae C38:1). Furthermore, as a planned sub-group analysis, we examined the correlation between the identified metabolite markers and vaginal bleeding. In this analysis there are no associations between early vaginal bleeding and the metabolite markers used in the placental abruption model.

**Table 3:** Area under the curve (AUC) of receiver operating characteristic (ROC) curves for placental abruption prediction.

	Area Under the ROC Curve	Model AUC Compared to VB	Error Rate
	(95% CI)	Model AUC	
**Model**			
Vaginal Bleeding Only	0.63 (0.55, 0.71)	—	0.37
Metabolites Only	0.68 (0.58, 0.79)	P = 0.480	0.36
Vaginal Bleeding + Metabolites	0.76 (0.66, 0.85)	P = 0.003	0.29

## Discussion

We identified metabolic differences between women predestined to develop abruption as compared to those whose pregnancies were not complicated by the disorder. Specifically, we identified alterations of fatty acid oxidation and phospholipid metabolism that may reflect abnormalities prior to the clinical presentation of abruption. Using quantitative metabolomics methods, we found that a sub-group of maternal serum metabolites (i.e., dodecanoylcarnitine/ dodecenoylcarnitine (C12 / C12:1), and phosphatidylcholine acyl-alkyl C 38:1 (PC ae C38:1) to be as predictive as the current clinical symptom of early pregnancy vaginal bleeding, and that the predictive probability of abruption was increased by using both the clinical symptom of vaginal bleeding and the metabolite biomarkers. The AUC for these metabolites alone was 0.68, for early pregnancy vaginal bleeding alone was 0.63, and combined the ACU significantly improved to 0.75 with the addition of quantitative metabolite data (P = 0.003).

Acylcarnitines, involved in organic acid metabolism, belong to a group of markers for mitochondrial function and reflect metabolic processes involved in long-chain fatty acid metabolism and related mitochondrial function [23]. They are synthesized by the enzyme carnitine palmitoyltransferase 1 (CPT 1) that is known to be responsible for the transport of fatty acids into the mitochondrial matrix.[24] Our analysis revealed significant differences in the concentrations of the acylcarnitine, dodecanoylcarnitine, which is present in fatty acid oxidation disorders such as long-chain acyl CoA dehydrogenase deficiency, carnitine palmitoyltransferase I deficiency, and carnitine palmitoyltransferase II deficiency.

Incomplete fatty acid oxidation results in elevated acylcarnitine concentrations [25], which is used in newborn screening to detect metabolic disorders.[26] Furthermore, alterations in concentrations of certain acylcarnitines have been documented in women with gestational diabetes mellitus [27] and in women with hypertensive disorders of pregnancy [28]. On balance, our observations are consistent with an emerging literature suggesting impaired mitochondrial function in the pathogenesis of abruption.

Available evidence suggests that impaired mitochondrial function may play a significant role in the complex pathogenesis of abruption [7,8,29,30]. In a pilot study of 233 abruption cases and 238 controls, Williams and colleagues [7] reported an increased odds of abruption with elevated mitochondrial DNA density (a biomarker of systemic mitochondrial dysfunction and oxidative stress) and that the association was particularly elevated among women with a concomitant diagnosis of preeclampsia and abruption. Namely, women with elevated mtDNA copy number and those with a diagnosis of preeclampsia had a 6.7-fold (95% CI 2.6–17.2) increased odds of abruption as compared with normotensive women without elevated mtDNA copy number. In addition, Qiu and colleagues[8] recently provided preliminary evidence for associations of target tissue-specific mitochondrial dysfunction with the risk abruption. Briefly, the authors reported that the odds of abruption was elevated among women who delivered placentas with higher mtDNA density (≥120.5, the median) as compared with those with lower values (<120.5) (OR = 2.4, 95% CI 1.1–5.1). These data are corroborated by findings from candidate gene studies that document elevated risks of abruption among women with variations in mitochondrial and oxidative phosphorylation related genes [29,30]. Our study showed that the odds of abruption increased with an increase in C12 / C12:1 and a decrease in PC ae C38:1. These related pathways (acylcarnitine or phosphaticholine) branch at the metabolite diacylglycerol [31]. Diacylglycerol is transformed to the endocannabinoid 2-arachidononylglycerol (2-AG), and 2-AG is converted to prostogladin glycerol esters. Recently Vaswani et al. [32] demonstrated that the enzyme prostaglandin-endoperoxide synthase-2 (PTGS-2), which converts 2-AG to prostaglandin glycerol esters, is down-regulated in the placenta, consistent with studies investigating the importance of PTGS-2 in preterm labor. These observations suggest that PTSGS-2 may play a role in the pathogenesis of abruption.

Several methodological strengths must be considered when interpreting the results of our study. First, our study provided preliminarily evidence for temporal relationship between maternal early pregnancy metabolomics profile and abruption risk later in pregnancy. Specifically, we documented alterations in maternal early pregnancy serum metabolomics profile (in samples collected 16 weeks gestation on average) several months before the condition was diagnosed clinically. Second, we were able to identify metabolites with both small and large case-control differences that are predictive of abruption. Third, in this initial study, we were able to combine clinical and quantitative metabolomics data to develop a clinical risk prediction model, which if confirmed in an independent sample, may have clinical utility. However, the small sample size our study hindered our capacity to formally assess statistical interactions of maternal characteristics (other than early pregnancy vaginal bleeding) and alteration in the early pregnancy serum metabolome on abruption risk. In a larger sample, we propose that investigation of statistical interactions of maternal metabolomics profile with other clinical characteristics such as maternal race/ethnicity, nutritional status and dietary intake, pre-pregnancy obesity, diabetes and hypertensive disorders may be informative. Our review of the available literature suggest that systematic evaluations of organic acid metabolites in relation to individual obesity status may be of etiologic importance. Future studies, with large sample sizes that allow for serial longitudinal measures of maternal serum metabolome across the trimesters of pregnancy are needed to further clarify the temporal relationship of specific organic acid metabolites and the incidence of abruption. Although we controlled for confounding, it cannot be concluded with certainty that the reported associations are unaffected by residual confounding. Of particular concern was the absence of detailed information about maternal use of illicit drugs during pregnancy, a previously identified placental abruption risk factor [4,33]. Finally, the stability of the metabolites after a long period of storage merits consideration. As part of the European project EnviroGenomarkers Hebels et al [34] studied the feasibility of using blood samples that had been stored in biobanks at -80°C for 4–19 years. Notably, the authors found that the long-term storage did not significantly affect the metabolomics analysis. Furthermore, we note that important scientific contributions have been made from metabolomics studies of archived samples including the Framingham Heart Study [35], the CARET study repository [36], and the Malmo Diet and Cancer Cardiovascular Cohort [37].

In summary, in this case-control metabolomics study of maternal early pregnancy serum samples, we identified fatty acids involved in organic acid metabolism, and choline-containing phospholipids, as well as a number of other compounds to be associated with the risk of incident abruption. Some of these candidate metabolites point towards a role of impaired mitochondrial function, and alterations in phospholipid and lipoprotein metabolism in abruption pathophysiology. We showed that several endogenous metabolites including long-chain acylcarnitines and phospholipids, in particular dodecanoylcarnitine, dodecenoylcarnitine and phosphatidylcholine acyl-alkyl C 38:1 are associated with incident abruption and that these metabolites, together with a clinical history of early pregnancy vaginal bleeding increase the predictive probability of the disorders. Future studies are warranted to further identify biomarkers of abruption and to elucidate the biological mechanisms of this important complication of pregnancy.

## References

[1] Oyelese Y, Ananth CV (2006) Placental abruption. Obstet Gynecol 108: 1005–1016. 17012465 10.1097/01.AOG.0000239439.04364.9a

[2] Ananth CV, Oyelese Y, Srinivas N, Yeo L, Vintzileos AM (2004) Preterm premature rupture of membranes, intrauterine infection, and oligohydramnios: risk factors for placental abruption. Obstet Gynecol 104: 71–77. 15229003 10.1097/01.AOG.0000128172.71408.a0

[3] Williams MA, Hickok DE, Zingheim RW, Luthy DA, Kimelman J, Nyberg DA, et al (1992) Elevated maternal serum alpha-fetoprotein levels and midtrimester placental abnormalities in relation to subsequent adverse pregnancy outcomes. Am J Obstet Gynecol 167: 1032–1037. 1384333 10.1016/s0002-9378(12)80033-0

[4] Williams MA, Lieberman E, Mittendorf R, Monson RR, Schoenbaum SC (1991) Risk factors for abruptio placentae. Am J Epidemiol 134: 965–972. 1951294 10.1093/oxfordjournals.aje.a116181

[5] Kramer MS, Usher RH, Pollack R, Boyd M, Usher S (1997) Etiologic determinants of abruptio placentae. Obstet Gynecol 89: 221–226. 9015024 10.1016/S0029-7844(96)00478-4

[6] Sanchez SE, Pacora PN, Farfan JH, Fernandez A, Qiu C, Aurora SK, et al (2006) Risk factors of abruptio placentae among Peruvian women. Am J Obstet Gynecol 194: 225–230. 16389036 10.1016/j.ajog.2005.05.013

[7] Williams MA, Sanchez SE, Ananth CV, Hevner K, Qiu C, et al (2013) Maternal blood mitochondrial DNA copy number and placental abruption risk: results from a preliminary study. Int J Mol Epidemiol Genet 4: 120–127. 23875065 PMC3709116

[8] Qiu C, Sanchez SE, Hevner K, Enquobahrie DA, Williams MA (2015) Placental mitochondrial DNA content and placental abruption: a pilot study. BMC Res Notes 8: 447 10.1186/s13104-015-1340-4 26377917 PMC4571073

[9] Arnold DL, Williams MA, Miller RS, Qiu C, Sorensen TK (2009) Iron deficiency anemia, cigarette smoking and risk of abruptio placentae. J Obstet Gynaecol Res 35: 446–452. 10.1111/j.1447-0756.2008.00980.x 19527381

[10] Tikkanen M, Hamalainen E, Nuutila M, Paavonen J, Ylikorkala O, Hiilesmaa V. (2007) Elevated maternal second-trimester serum alpha-fetoprotein as a risk factor for placental abruption. Prenat Diagn 27: 240–243. 17238224 10.1002/pd.1654

[11] Yaron Y, Heifetz S, Ochshorn Y, Lehavi O, Orr-Urtreger A (2002) Decreased first trimester PAPP-A is a predictor of adverse pregnancy outcome. Prenat Diagn 22: 778–782. 12224070 10.1002/pd.407

[12] Williams MA, Hickok DE, Zingheim RW, Zebelman AM (1993) Maternal serum CA 125 levels in the diagnosis of abruptio placentae. Obstet Gynecol 82: 808–812. 8414329

[13] Signore C, Mills JL, Qian C, Yu KF, Rana S, Karumanchi SA, et al (2008) Circulating soluble endoglin and placental abruption. Prenat Diagn 28: 852–858. 10.1002/pd.2065 18702104 PMC2574843

[14] Signore C, Mills JL, Qian C, Yu K, Lam C, et al (2006) Circulating angiogenic factors and placental abruption. Obstet Gynecol 108: 338–344. 16880304 10.1097/01.AOG.0000216014.72503.09

[15] Onalan R, Onalan G, Gunenc Z, Karabulut E (2006) Combining 2nd-trimester maternal serum homocysteine levels and uterine artery Doppler for prediction of preeclampsia and isolated intrauterine growth restriction. Gynecol Obstet Invest 61: 142–148. 16374017 10.1159/000090432

[16] Nilsen RM, Vollset SE, Rasmussen SA, Ueland PM, Daltveit AK (2008) Folic acid and multivitamin supplement use and risk of placental abruption: a population-based registry study. Am J Epidemiol 167: 867–874. 10.1093/aje/kwm373 18187445

[17] Romero R, Mazaki-Tovi S, Vaisbuch E, Kusanovic JP, Chaiworapongsa T, Gomez R, et al (2010) Metabolomics in premature labor: a novel approach to identify patients at risk for preterm delivery. J Matern Fetal Neonatal Med 23: 1344–1359. 10.3109/14767058.2010.482618 20504069 PMC3440243

[18] Austdal M, Skrastad RB, Gundersen AS, Austgulen R, Iversen AC, Bathen TF. (2014) Metabolomic biomarkers in serum and urine in women with preeclampsia. PLoS One 9: e91923 10.1371/journal.pone.0091923 24637620 PMC3956817

[19] Horgan RP, Broadhurst DI, Dunn WB, Brown M, Heazell AE, Kell DB, et al (2010) Changes in the metabolic footprint of placental explant-conditioned medium cultured in different oxygen tensions from placentas of small for gestational age and normal pregnancies. Placenta 31: 893–901. 10.1016/j.placenta.2010.07.002 20708797

[20] Enquobahrie DA, Denis M, Tadesse MG, Gelaye B, Ressom HW, Williams MA. (2015) Maternal Early Pregnancy Serum Metabolites and Risk of Gestational Diabetes Mellitus. J Clin Endocrinol Metab 100: 4348–4356. 10.1210/jc.2015-2862 26406294 PMC4702451

[21] Xiao R, Sorensen TK, Frederick IO, El-Bastawissi A, King IB, Leisenring WM, et al (2002) Maternal second-trimester serum ferritin concentrations and subsequent risk of preterm delivery. Paediatr Perinat Epidemiol 16: 297–304. 12445145 10.1046/j.1365-3016.2002.00448.x

[22] Zukunft S, Sorgenfrei M, Prehn C, Möller G, Adamski J (2013) Targeted Metabolomics of Dried Blood Spot Extracts. Chromatographia 76: 1295–1305.

[23] Lord R, Bralley J (2008) Laboratory evaluations for integrative and functional medicine. 2nd ed. Duluth, GA: Metametrix Institution.

[24] Indiveri C, Iacobazzi V, Tonazzi A, Giangregorio N, Infantino V, Convertini P, et al (2011) The mitochondrial carnitine/acylcarnitine carrier: function, structure and physiopathology. Mol Aspects Med 32: 223–233. 10.1016/j.mam.2011.10.008 22020112

[25] Koves TR, Noland RC, Bates AL, Henes ST, Muoio DM, et al (2005) Subsarcolemmal and intermyofibrillar mitochondria play distinct roles in regulating skeletal muscle fatty acid metabolism. Am J Physiol Cell Physiol 288: C1074–1082. 15647392 10.1152/ajpcell.00391.2004

[26] Van Hove JL, Zhang W, Kahler SG, Roe CR, Chen YT, Cortright RN. (1993) Medium-chain acyl-CoA dehydrogenase (MCAD) deficiency: diagnosis by acylcarnitine analysis in blood. Am J Hum Genet 52: 958–966. 8488845 PMC1682046

[27] Qiu C, Enquobahrie DA, Frederick IO, Sorensen TK, Fernandez MA, David RM, et al (2014) Early pregnancy urinary biomarkers of fatty acid and carbohydrate metabolism in pregnancies complicated by gestational diabetes. Diabetes Res Clin Pract 104: 393–400. 10.1016/j.diabres.2014.03.001 24703806 PMC4077203

[28] Koster MP, Vreeken RJ, Harms AC, Dane AD, Kuc S, Schielen PC, et al (2015) First-Trimester Serum Acylcarnitine Levels to Predict Preeclampsia: A Metabolomics Approach. Dis Markers 2015: 857108 10.1155/2015/857108 26146448 PMC4471382

[29] Denis M, Enquobahrie DA, Tadesse MG, Gelaye B, Sanchez SE, Salazar M, et al (2014) Placental genome and maternal-placental genetic interactions: a genome-wide and candidate gene association study of placental abruption. PLoS One 9: e116346 10.1371/journal.pone.0116346 25549360 PMC4280220

[30] Workalemahu T, Enquobahrie DA, Moore A, Sanchez SE, Ananth CV, Pacora PN, et al (2013) Genome-wide and candidate gene association studies of placental abruption. Int J Mol Epidemiol Genet 4: 128–139. 24046805 PMC3773564

[31] Seibel BA, Walsh PJ (2002) Trimethylamine oxide accumulation in marine animals: relationship to acylglycerol storage. J Exp Biol 205: 297–306. 11854367 10.1242/jeb.205.3.297

[32] Vaswani K, Chan HW, Peiris HN, Nitert MD, Bradley RJ, Armitage JA, et al (2015) Gestation Related Gene Expression of the Endocannabinoid Pathway in Rat Placenta. Mediators Inflamm 2015: 850471 10.1155/2015/850471 26229240 PMC4503552

[33] Kistin N, Handler A, Davis F, Ferre C (1996) Cocaine and cigarettes: a comparison of risks. Paediatr Perinat Epidemiol 10: 269–278. 8822770 10.1111/j.1365-3016.1996.tb00050.x

[34] Hebels DG, Georgiadis P, Keun HC, Athersuch TJ, Vineis P, Vermeulen R, et al (2013) Performance in omics analyses of blood samples in long-term storage: opportunities for the exploitation of existing biobanks in environmental health research. Environ Health Perspect 121: 480–487. 10.1289/ehp.1205657 23384616 PMC3620742

[35] Wang TJ, Ngo D, Psychogios N, Dejam A, Larson MG, Vasan RS, et al (2013) 2-Aminoadipic acid is a biomarker for diabetes risk. J Clin Invest 123: 4309–4317. 10.1172/JCI64801 24091325 PMC3784523

[36] Wikoff WR, Hanash S, DeFelice B, Miyamoto S, Barnett M, Zhao Y, et al (2015) Diacetylspermine Is a Novel Prediagnostic Serum Biomarker for Non-Small-Cell Lung Cancer and Has Additive Performance With Pro-Surfactant Protein B. J Clin Oncol 33: 3880–3886. 10.1200/JCO.2015.61.7779 26282655 PMC4652011

[37] Magnusson M, Lewis GD, Ericson U, Orho-Melander M, Hedblad B, Engström G, et al (2013) A diabetes-predictive amino acid score and future cardiovascular disease. Eur Heart J 34: 1982–1989. 10.1093/eurheartj/ehs424 23242195 PMC3703309

